# Retraction notice to "Development data associated with effects of stiffness softening of 3D-TIPS elastomer nanohybrid scaffolds on tissue ingrowth, vascularization and inflammation in vivo” [Data in Brief 22(2019) 885 - 902]

**DOI:** 10.1016/j.dib.2026.112674

**Published:** 2026-03-18

**Authors:** Linxiao Wu, Adrián Magaz, Elizabeth Maughan, Nina Oliver, Arnold Darbyshire, Marilena Loizidou, Mark Emberton, Martin Birchall, Wenhui Song

**Affiliations:** a Centre for Biomaterials in Surgical Reconstruction and Regeneration, Division of Surgery & Interventional Science, University College London, London NW3 2PF, United Kingdom; b UCL Ear Institute, Royal National Throat Nose and Ear Hospital and University College London, London, United Kingdom

This article has been retracted: please see Elsevier Policy on Article Withdrawal (https://www.elsevier.com/about/policies/article-withdrawal).

This article has been retracted at the request of the Editor-in-Chief and Editor.

Concerns were raised regarding potential duplication and manipulation of several figure panels in the article. The authors were invited to respond and provided some explanations and revised legends; however, the concerns were not adequately resolved. Specifically, duplicated and/or manipulated images were observed in multiple figure panels (including but not limited to Figures 4J/5D; 7H/8G; 8H/9H; 9H/10H; 11J/12K), with no verifiable raw data provided to support the published results. The inability to supply the original data, combined with inconsistencies in the authors’ explanations, undermines the reliability of the findings presented.

The authors did not respond satisfactorily to the journal’s final request for clarification. As a result, the Editors no longer have confidence in the integrity of the article’s content.

The journal was able to contact authors W.Song, L.Wu, E.Maughan, M.Louzidou, M.Emberton and M.Birchall, but was unable to contact authors N.Oliver, A.Darbyshire and A.Magaz.

